# A comparative study on roles of natural killer T cells in two diet-induced non-alcoholic steatohepatitis-related fibrosis in mice

**DOI:** 10.1080/07853890.2022.2108894

**Published:** 2022-08-11

**Authors:** Shumei Zheng, Wenzhuo Yang, Dongmei Yao, Shanhong Tang, Juanni Hou, Xing Chang

**Affiliations:** a Department of Gastroenterology and Hepatology, The General Hospital of Western Theater Command, Chengdu, China; b Department of Gastroenterology and Hepatology, Shanghai Tongji Hospital, Shanghai Tongji University, Shanghai, China; c Department of Gastroenterology and Hepatology, the Second Hospital of Hebei Medical University, Shijiazhuang, China

**Keywords:** Natural killer T cell, non-alcoholic fatty liver disease, cytokine, α-galactosylceramide

## Abstract

**Background:**

Immune responses are important in the progression of non-alcoholic fatty liver disease (NAFLD). Natural killer T (NKT) cells are main components of the innate immune system that modulate immunity. However, the role of NKT cells in NAFLD remains controversial.

**Objective:**

We aimed to investigate the role of NKT cells in non-alcoholic steatohepatitis (NASH)-related fibrosis in fast food diet (FFD)- and methionine choline-deficient (MCD) diet-induced mouse models.

**Methods:**

Hepatic NKT cells were analysed in wild-type (WT) and CD1d-/- mice fed FFD or MCD diets. Hepatic pathology, cytokine profiles and liver fibrosis were evaluated. Furthermore, the effect of chronic administration of α-galactosylceramide (α-GalCer) on liver fibrosis was investigated in both FFD- and MCD-treated mice.

**Results:**

FFD induced a significant depletion of hepatic NKT cells, thus leading to mild to moderate NASH and early-stage fibrosis, while mice fed MCD diets developed severe liver inflammation and progressive fibrosis without a significant change in hepatic NKT cell abundance. FFD induced a similar liver fibrogenic response in CD1d-/- and WT mice, while MCD induced a higher hepatic mRNA expression of Col1α1 and TIMP1 as well as relative fibrosis density in CD1d-/- mice than WT mice (31.8 *vs*. 16.3, *p* = .039; 40.0 *vs*. 22.6, *p* = .019; 2.24 *vs*. 1.59, *p* = .036). Chronic administration of α-GalCer induced a higher hepatic mRNA expression of TIMP1 in MCD-treated mice than controls (36.7 *vs.* 14.9, *p* = .005).

**Conclusion:**

NKT cells have protective roles in NAFLD as the disease progresses. During diet-induced steatosis, mild to moderate NASH and the early stage of fibrosis, hepatic NKT cells are relatively depleted, leading to a proinflammatory status. In severe NASH and the advanced stage of liver fibrosis, NKT cells play a role in inhibiting the NASH-related fibrogenic response. Chronic administration of α-GalCer induces NKT cell anergy and tolerance, which may play a role in promoting the liver fibrogenic response.

## Introduction

NAFLD ranges from simple steatosis to NASH, which can progress to cirrhosis and hepatocellular carcinoma. Compared to individuals with simple steatosis, individuals with NASH are more likely to develop advanced stages of liver fibrosis, cirrhosis and carcinoma. Although the mechanisms that drive disease progression remain uncertain, accumulating evidence suggests that the immune system may participate in this process [1,2].

More recently, increasing emphasis has been placed on altered innate immunity as a key event in the development of inflammation in NAFLD. The liver contains enriched innate immune cells, including Kupffer cells (KCs), NK cells and NKT cells, possibly because of a defence mechanism against constant exposure to a variety of toxins and antigens from intestinal bacteria through portal veins. The role of immune cells on NAFLD has yet to be fully elucidated. The population of NK cells is enriched and accounts for 5%–10% of hepatic lymphocytes in mice [3]. In a previous study, Theurich and co-authors indicate that obesity-associated inflammation and metabolic disturbances depend on IL-6/Stat 3-dependent formation of a distinct NK population in high-fat diet (HFD)-induced obesity in mice [4]. Conversly, Fan and co-authors demonstrate that intrahepatic NK cells, recruited through CXCL10-CXCR3 interaction, play a protective role against the fibrosis progression in MCD-induced NASH in mice [3]. NKT cells, another population of innate immune cells, are most abundant in the liver, accounting for 20%-35% of total mouse hepatic lymphocytes [5]. NKT cells specifically recognise glycolipid antigens, such as α-GalCer, which is presented by the MHC classI-like molecule CD1d, and can produce both Th1 and Th2 cytokines when activated [5–7]. However, the role of NKT cells in hepatic fibrogenesis is poorly understood.

The ability of NKT cells to modulate disease progression in NAFLD has been an interesting issue because these cells are activated by lipid antigens, and NAFLD is a disorder of fat homeostasis. A previous study showed hepatic NKT cell depletion in leptin-deficient ob/ob mice, a model of obesity-related proinflammatory cytokine excess, insulin resistance and NASH [8,9]. Hepatic NKT cells were depleted when WT mice were administered high-fat and high-sucrose diets, leading to proinflammatory cytokine polarisation [10,11]. Adoptive transfer of NKT cells to ob/ob mice and HFD mice ameliorated steatohepatitis and improved glucose homeostasis [10,12]. Conversely, an accumulation of hepatic NKT cells in MCD-induced NAFLD in mice has been reported [13]. Liver fibrosis was attenuated in CD1d-/- mice that lacked NKT cells [13,14]. In addition, Maricic and his colleagues indicate that activation of invariant NKT cells play a key role in mediating diet-induced hepatic steatosis and fibrosis in mice and its potential involvement in NASH progression in humans [15].

Here, we developed two models of diet-induced liver fibrosis in mice to investigate the role of hepatic NKT cells in different microenvironments of liver fibrosis. The MCD model is one of the most common dietary NAFLD models accompanied by steatohepatitis and progressive fibrosis in mice, but in a metabolic context, it is distinct from that of humans with NASH [16,17]. More recently, another rodent model, the FFD model, involving a high fat, high cholesterol and high fructose diet with a sedentary lifestyle, appeared to be a new NAFLD model that may represent human metabolic syndrome (MS) and NASH with fibrosis [18]. We fed WT and CD1d-/- mice either an FFD or MCD diet to evaluate whether NKT cells modulate NASH-related fibrosis and whether chronic administration of α-GalCer, an NKT cell activator, has an effect on liver fibrogenesis.

## Materials and methods

### Animal experiments

**Figure 1.**
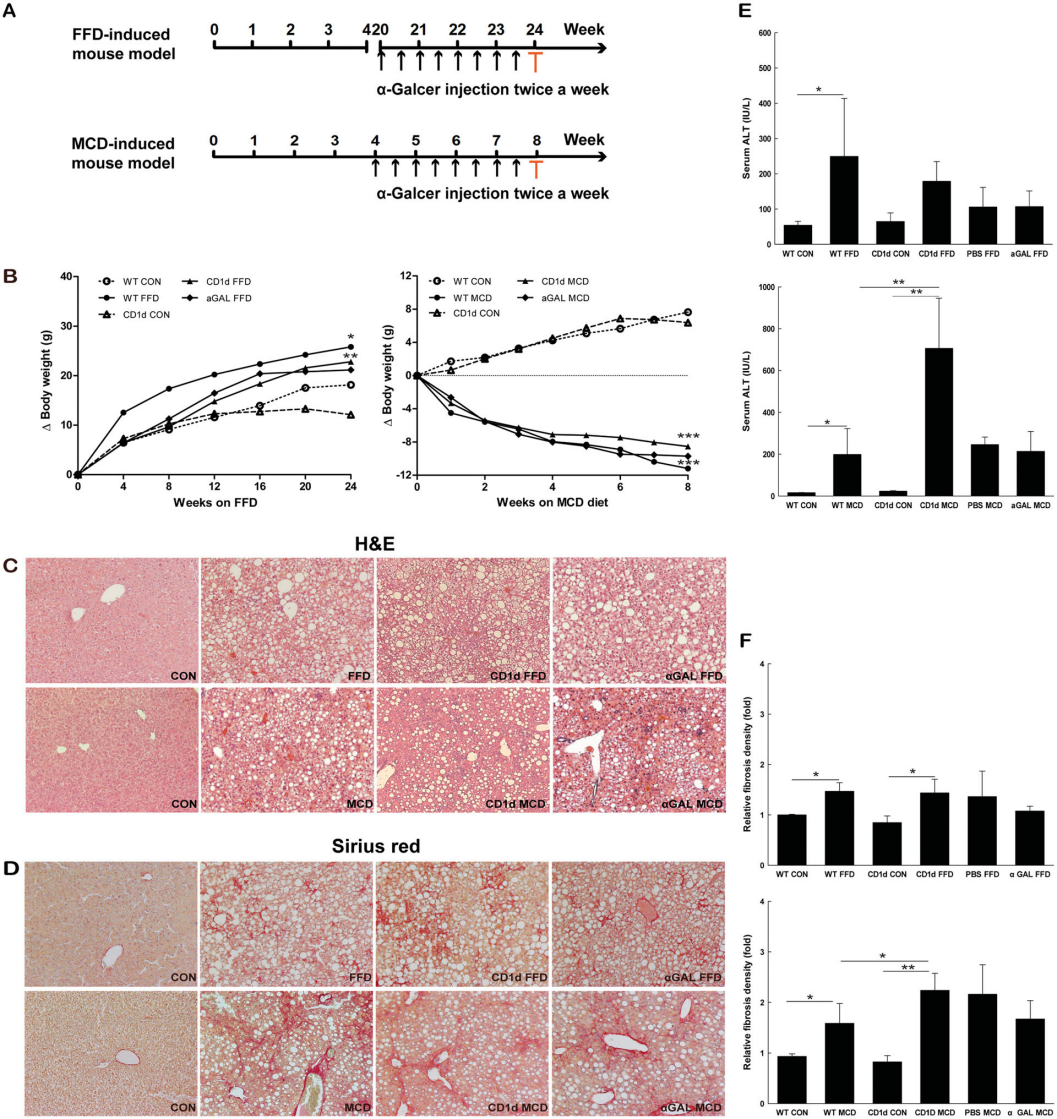
The MCD diet induces a higher degree of NASH and more advanced liver fibrosis than FFD in mice WT and CD1d-/- mice were fed normal diet, FFD, MCD diet, or FFD or MCD diet plus chronic intraperitoneal injection of α-GalCer (1.6 μg/mouse, twice a week for the last 4 weeks). (A) Study design of FFD- and MCD-induced mouse models. The sera and liver tissues were collected at the end of the experiment highlighted by red colour. (B) Gain of body weight in WT or CD1d-/- mice fed normal diet, FFD or MCD diet, (C) Hematoxylin-Eosin staining, (D) Sirius red staining (10 × magnification), (E) ALT levels and (F) Fibrosis quantification by morphometric analysis were performed. The results of fibrosis quantification were expressed as fold change relative to WT control chow-fed mice (*n* = 5 per group). *p <.05, **p < .01 *versus* the respective control group.

Adult male (6–8 week) wild-type (WT) C57BL/6 (Bar Harbour, ME) and CD1d-deficient mice on C57BL/6 background (Bar Harbour, ME) were fed either MCD diet for 8 weeks or FFD, providing 40% of energy as fat (milk fat, 12% saturated) with 2% cholesterol (AIN-76A.W.D) for 24 weeks (Figure 1(A)) (*n* = 5 mice per subgroup). Mice fed FFD were fed special water that a total of 42 g/L of carbohydrates was mixed in drinking water at a ratio of 55% fructose (Sigma) and 45% sucrose (Sigma) by weight. MCD diet were permitted *ad libitum* consumption of water, and those fed control diet were fed standard chow diet, providing 13% of energy as fat (milk fat, 0.9% saturated). A stock solution of α–GalCer (Alexis Biochemicals Corp.) was diluted to 0.2 mg/200 ml in 0.5% polysorbate-20 and stored at −20 °C. Chronic administration of α-GalCer (1.6 μg/200 μL in phosphate-buffered saline per mouse) was carried out by intraperitoneal injection twice per week for the last four weeks (Figure 1(A)). There was no mortality in mice treated with diets and α -GalCer. All mice were kept on a 12:12-hour light-dark schedule at 23 °C. All the animal experiments were followed protocols approved by the Institutional Animal Care and Treatment Committee at the General Hospital of Western Theater Command (Reference number: 2021EC4-81), and also followed the ARRIVE guidelines.

### Liver histology

The liver sections imbedded in paraffin were cut (5 μm) and stained with Hematoxylin-Eosin or Sirius red as described previously. Ten × 100 light-microscopic fields were assessed on each section and scored for the extent of steatosis and 10 × 200 fields for inflammation score according to the criteria described previously [19]. The histology was evaluated by an experienced hepatopathologist. Liver fibrosis was determined by measuring the intensity of fibrosis in 10 × 100 digital images captured from slides of each mouse stained with Sirius red using MetaMorph 6.0 imaging software (Molecular Devices Co.). The total fibrosis density score was determined by dividing the image intensity by the image area as described previously [20].

### Hepatic mononuclear cell isolation and cell surface labelling

Hepatic mononuclear cells (HMNCs) were isolated as previously described [8]. After isolation, HMNCs were labelled with CD1d-Tetramers loaded with a ligand (PBS-57, an analogue of α-galactosylceramide) or anti-mouse fluorescent antibodies against NK1.1 and CD3, CD4, and CD8 (Pharmingen). After surface labelling, HMNCs were evaluated by flow cytometry (Becton Dickinson). Data were analysed by Cell Quest software (Becton Dickinson).

### Hepatic mononuclear cell intracellular cytokines labelling

For intracellular staining of cytokines, HMNCs were incubated with phorbol 1,2-myristate 1,3-acetate (Sigma, 50 ng/ml), ionomycin (Sigma, 500 ng/ml), and GolgiPlug (Pharmingen, 1 μl/ml). Cells were labelled with surface antibodies and then permeabilized with Cytoperm/Cytofix (Pharmingen) according to the manufacturer’s instruction. After permeabilization, cells were further labelled with anti-mouse interleukin IL-4 and IFN-γ (Pharmingen), and then evaluated by flow cytometry. Data were analysed as described in previous paragraph.

### Determination of messenger RNA by real-time quantitative polymerase chain

The 7900 HT and the SDS 2.2.1 software were used to perform real-time quantitative polymerase chain reaction (RT-qPCR). Total RNA was obtained from liver tissues and cocultured with TRIzol reagent (Invitrogen). The concentration of the isolated RNA was determined from the optical density at 260 nm and its purity from the 260/280 nm OD ratio. The RNA was reverse-transcribed with Primescript RT Reagent kit (TaKaRa, Japan) according to manufacture specifications. The mean value of the triplicate for each sample was calculated and expressed as cycle threshold (CT). The amount of gene expression was then calculated as the difference (δCT) between the CT value of the sample for the target gene and the mean CT value of the endogenous control (GAPDH). The relative level of expression was measured as 2^-δδCT^.

### Statistical analysis

All values were expressed as mean ± SD. The group means were compared by ANOVA, using SPSS 21.0 software. Statistical significance was considered at *p* < .05.

## Results

### The FFD increases a body weight, while the MCD diet induces a body weight loss in mice

Adult male C57BL6 mice were fed either FFD or MCD diets. The body weight of these mice was monitored every week after the initiation of feeding. The FFD increased the body weight by around 100% at week 24, while the MCD diet induced a body weight loss by around 40% at week 8 in both WT and CD1d-/- mice. There is no significant difference of body weight change between FFD or MCD diet-treated WT and CD1d-/- mice (Figure 1(B)). We examined the effects of repeated α-GalCer intraperitoneal injection on body weight in FFD- and MCD-fed WT mice. The results showed it did not affect body weight in both groups (Figure 1(B)).

### The MCD diet induces a higher degree of NASH and more advanced liver fibrosis than FFD

To investigate the role of NKT cells in the development of NASH-related fibrosis, both WT and CD1d-/- mice were fed either FFD or MCD diets. As expected, mice fed FFD developed microvesicular and macrovesicular steatosis, hepatocellular ballooning, intraacinar inflammation commonly associated with mild to moderate NASH and stage 1 fibrosis, which mainly occurred in a perisinusoidal or pericellular pattern (Table 1, Figure 1(C,D)). MCD mice developed paracinar macrovesicular steatosis, severe inflammation, hepatocellular ballooning commonly associated with severe NASH and more advanced stage of fibrosis (mostly stage 2 or stage 3), which occurred in a perisinusoidal, pericellular, perivenular or bridging fibrosis pattern (Table 1, Figure 1(C,D)). The MCD diet induced a higher degree of NASH (Figure 1(C)) and a more advanced stage of liver fibrosis than FFD in either WT or CD1d-/- mice (Table 1, Figure 1(D)).

**Table 1.** Histological scores of livers from FFD and MCD mice.

Mean (SD)	FFD					MCD				
	WT con	WT FFD	CD1d con	CD1d FFD	αGal FFD	WT con	WT MCD	CD1d con	CD1d MCD	αGal MCD
**Steatosis**	0 (0)	2.58 (0.08)**	0 (0)	2.76 (0.17)**	2.74 (0.18)	0 (0)	2.78 (0.12)**	0 (0)	2.75 (0.24)**	2.83 (0.21)
**Inflammation**	0 (0)	1.05 (0.08)**	0 (0)	1.20 (0.55)**	1.12 (0.28)	0 (0)	2.67 (0.58)**	0 (0)	2.67 (0.58)**	2.58 (0.52)
**Ballooning**	0 (0)	0.88 (0.34)*	0 (0)	0.99 (0.42)*	0.84 (0.53)	0 (0)	1.88 (0.34)**	0 (0)	1.76 (0.22)**	1.68 (0.23)
**Fibrosis**	0 (0)	0.91 (0.13)*	0 (0)	0.89 (0.23)*	0.86 (0.34)	0 (0)	2.76 (0.51)**	0 (0)	2.81 (0.47)**	2.87 (0.35)

Mean values (±SD) for mice are shown. Steatosis was graded as 0 (none), 1 (<33% HPF), 2 (33–66% HPF), or 3 (>66% HPF). Inflammation was graded as 0 (none), 1 (<2 foci/HPF), 2 (2-4 foci/HPF), or 3 (>4 foci/HPF). Hepatocyte ballooning was graded as 0 (none), 1 (few balloon cells), or 2 (many). Fibrosis was graded as 0 (none), 1 (perisinusoidal or periportal fibrosis), 2 (perisinusoidal and portal/periportal fibrosis), 3 (bridging fibrosis), or 4 (cirrhosis). *p < .05, **p < .001 *versus* the respective controls.

CD1d deficiency exacerbated liver injury in mice fed MCD diets (706.4 *vs*. 199.1, *p* = .003), while it had no effect on ALT levels in mice fed FFD (178.9 *vs.* 249.3, *p* = .276) (Figure 1(E)), which were lacking in hepatic NKT cells, suggesting an antiinflammatory role of NKT cells in liver injury. Furthermore, chronic intraperitoneal injection of α-GalCer had little effect on the ALT level in FFD and MCD mice (106.9 *vs*. 106.2, *p* = .993; 213.6 *vs*. 246.0, *p* = .747) (Figure 1(E)).

To better understand the role of NKT cells in NASH-related fibrosis, we subsequently investigated whether CD1d deficiency had an effect on the relative fibrosis quantity in both FFD- and MCD-induced fibrosis. The area of hepatic fibrosis detected by Sirius red staining and densitometric analysis showed a higher relative fibrosis density in CD1d-/- mice than WT mice fed MCD diets (2.2 *vs.* 1.6, *p* =.036), while no morphological change was observed between FFD-treated WT and CD1d-/- mice (1.4 *vs*. 1.5, *p* = .873) (Figure 1(F)). In addition, chronic intraperitoneal injection of α-GalCer had little effect on the relative fibrosis quantity in FFD or MCD mice (1.1 *vs*. 1.4, *p* = .192; 1.7 *vs.* 2.2, *p* = .101) (Figure 1(F)).

### FFD causes hepatic NKT cell depletion, while MCD diet induces an insignificant change in NKT cell abundance

**Figure 2.**
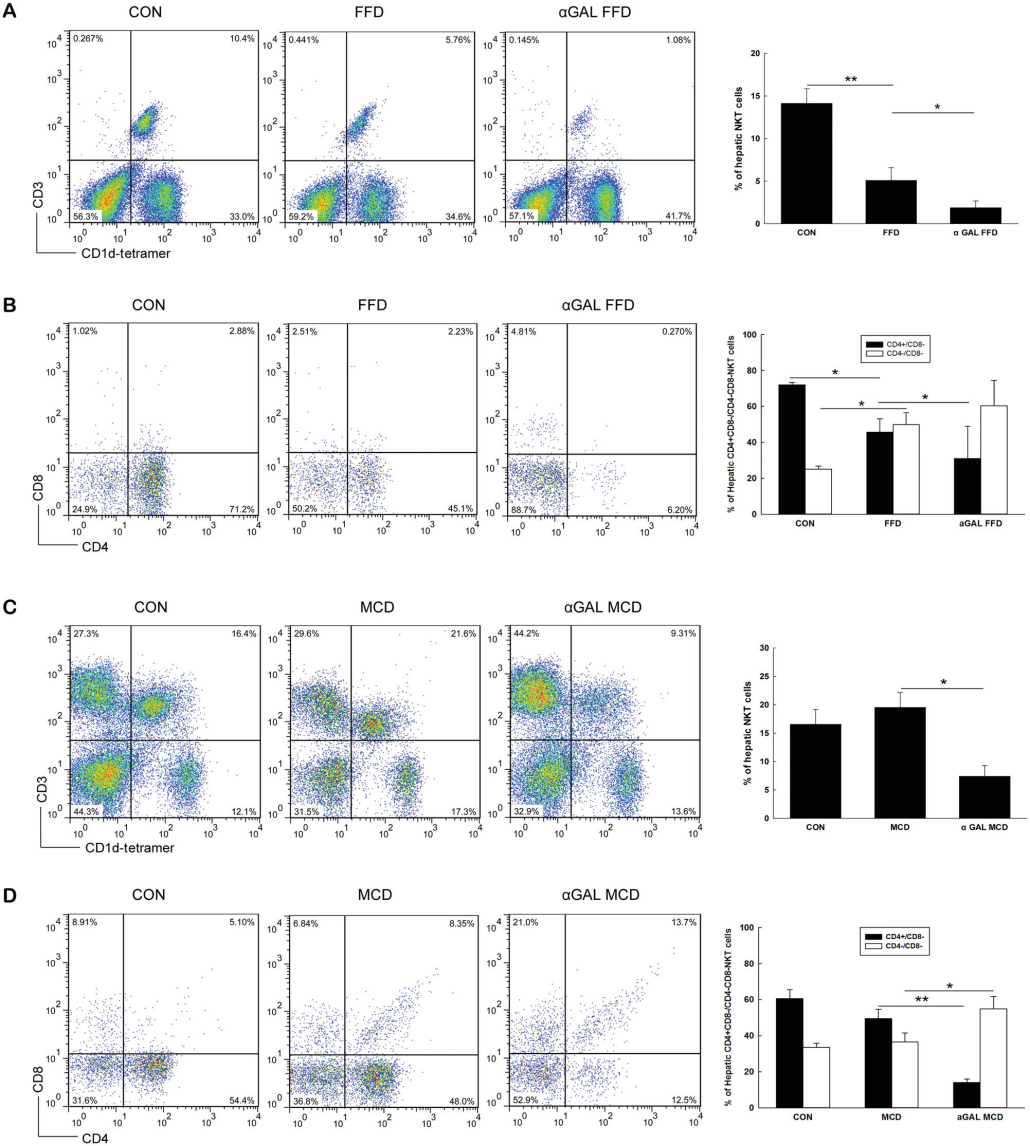
FFD causes hepatic NKT cell depletion, while MCD diet induces an insignificant change in NKT cell abundance in WT mice WT mice were fed normal diet, FFD, or MCD diet with or without chronic administration of α-GalCer (*n* = 3 per group). Hepatic mononuclear cells were isolated and total hepatic NKT cells and CD4 + NKT cell subpopulations were identified. (A or C) Dot plot of hepatic CD3 and CD1d tetramer positive cells and results of hepatic NKT percentages in FFD (A) or MCD (C) groups. (B or D) Percentages of hepatic CD4 + NKT cell subpopulations in FFD (B) or MCD (D) groups. *p < .05, **p < .01.

We then evaluated HMNC subsets using cell surface markers and flow cytometry. As shown in Figure 2(A), mice fed FFD had significantly fewer hepatic NKT cells than those in the normal diet group (5.1 *vs*. 14.1%, *p* = .005). The decrease in liver NKT cells was predominantly attributable to reduced numbers of CD4^+^/CD8^-^ NKT cells in FFD mice (45.8 *vs*. 72.0%, *p* = .010) (Figure 2(B)). Unlike the FFD group, mice fed the MCD diet showed no significant change in hepatic NKT cell abundance compared to the normal diet group (20.0 *vs*. 16.6%, *p* = .153) (Figure 2(C)). After chronic injection of α-GalCer, the proportion of NKT cells either in FFD mice or in the MCD diet group significantly decreased compared to the controls (1.9 *vs*. 5.1%, *p* = .036; 7.4 *vs*. 20.0%, *p* = .017) (Figure 2(A,C)). Further analysis indicated that the decrease in NKT cells after chronic injection of α-GalCer was predominantly attributable to reduced numbers of CD4^+^/CD8^-^ NKT cells in either FFD or MCD mice (31.1 *vs*. 45.8%, *p* = .023; 14.1 *vs*. 49.5%, *p* = .001) (Figure 2(B,D)).

### Both FFD and MCD diets increase hepatic proinflammatory cytokine production

**Figure 3.**
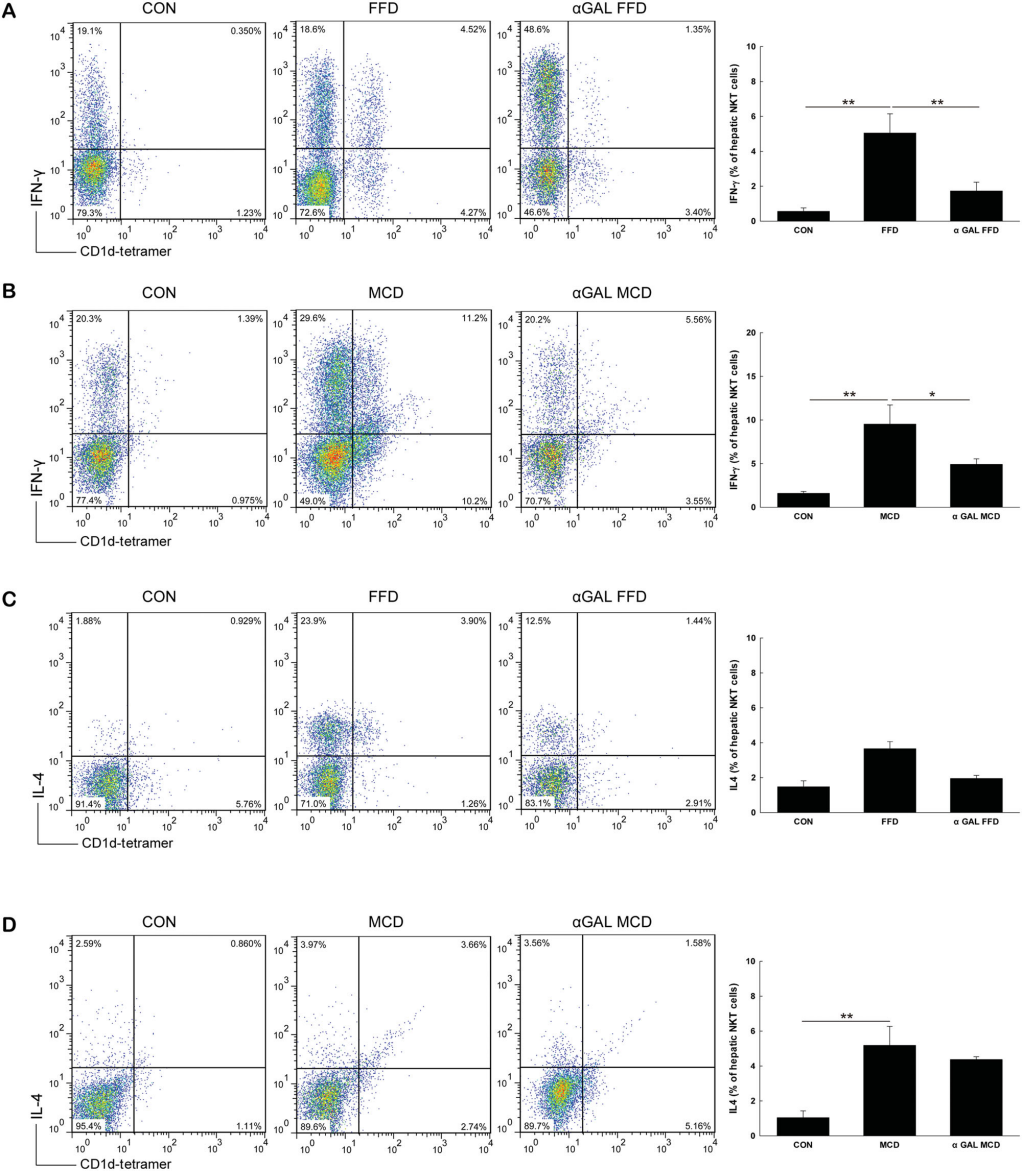
FFD increases intracellular Th1 cytokine production, while MCD diet increases both Th1 and Th2 cytokine from hepatic NKT cells Mice were treated the same as described in Figure 2. Hepatic mononuclear cells were isolated and total NKT cells were identified with CD1d tetramer loaded with a ligand and colabeled with CD3, IFN-γ and IL-4. The percentages of IFN-γ and IL-4 positive hepatic NKT cells from FFD (A and C) and MCD (B and D) mice are presented as both dot plot and mean ± SD values (*n* = 3 per group). *p <.05, **p < .01.

We next investigated the effect of both FFD and MCD diets on hepatic NKT cell cytokine profiles. As shown in Figure 3(A and B), both FFD and MCD diets significantly increased intracellular IFN-γ production from hepatic NKT cells compared with control mice (5.1 *vs.* 0.6, *p* = .003; 9.5 *vs*. 1.6, *p* = .006). The level of intracellular IL-4 from hepatic NKT cells was insignificantly changed in the FFD group (3.7 *vs*. 1.5, *p* = .177) (Figure 3(C)) but was significantly elevated in mice fed MCD diets compared to the normal diet groups (5.2 *vs*. 1.0, *p* = .001) (Figure 3(D)), suggesting intracellular Th1 polarization of cytokines from hepatic NKT cells in the FFD group and increases in both Th1 and Th2 cytokines in MCD mice.

**Figure 4.**
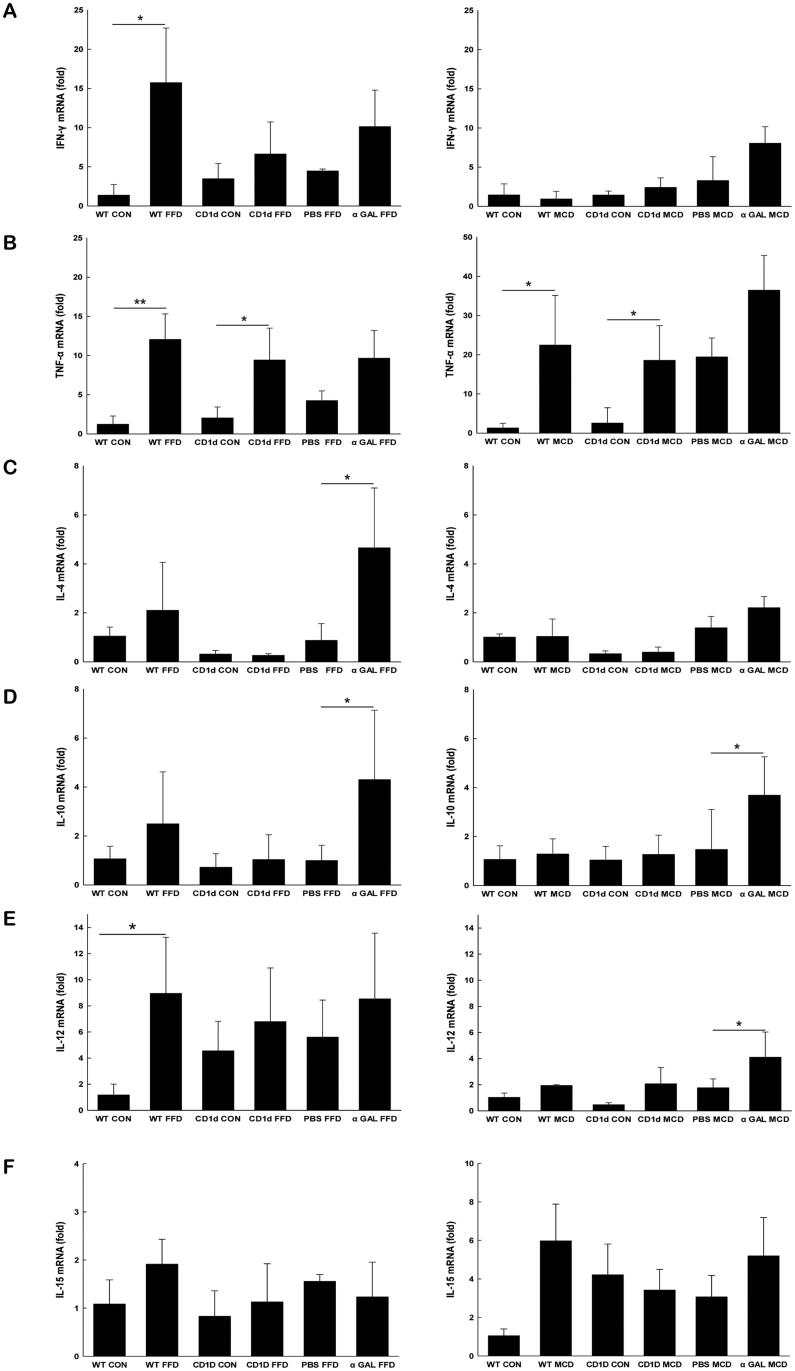
Both FFD and MCD diets increase hepatic proinflammatory cytokine production Hepatic mRNA expression of IFN-γ(A), TNF-α(B), IL-4 (C), IL-10 (D), IL-12 (E) and IL-15 (F) was detected by RT-qPCR. The data are presented as mean ± SD (*n* = 5 per group). *p < .05, **p < .01.

To evaluate cytokine production at the whole liver level, we next examined the hepatic mRNA expression of cytokines. The result showed that IFN-γ significantly increased in WT mice than control mice reared on FFD (15.8 *vs*. 1.4, *p* = .043) (Figure 4(A)), and TNF-α was obviously elevated in both WT and CD1d-/- mice fed either FFD or MCD diet compared to the respective controls (12.1 *vs*. 1.2, *p* = .006; 9.4 *vs*. 2.0, *p* = .042; 22.5 *vs*. 1.3, *p* = .040; 18.6 *vs*. 2.5, *p* = .045) (Figure 4(B)), suggesting Th1 polarisation of the hepatic cytokine profile in both the FFD and MCD groups.

It has been reported that chronic α-GalCer administration may lead to NKT cell anergy, tolerance, and imbalance of Th1 and Th2 cytokine profiles. Our subsequent efforts were directed to determine whether repeated α-GalCer injection has an effect on hepatic cytokine production. The data showed that after repeated α-GalCer injection, IFN-γ production from NKT cells in either FFD or MCD mice was profoundly blunted (1.7 *vs*. 5.1, *p* =.006; 4.9 *vs*. 9.5, *p* = .010) (Figure 3(A,B)), while levels of IL-4 from NKT cells were insignificantly changed compared to the respective control (2.0 *vs*. 3.7, *p* = .243; 4.4 *vs.* 5.2, *p* = .343) (Figure 3(C,D)), suggesting a predominate Th2 polarisation in hepatic NKT cells in both FFD and MCD mice. Further investigation indicated that chronic α-GalCer administration significantly increased hepatic IL-4 (4.7 *vs*. 0.9, *p* = .021) (Figure 4(C)) and IL-10 mRNA levels (4.3 *vs*. 0.9, *p* =.013) (Figure 4(D)) in FFD mice and upregulated IL-10 mRNA expression in MCD mice compared to the control (3.7 *vs*. 1.5, *p* = .014) (Figure 4(D)), also suggesting Th2 cytokine profiles at the whole liver level after chronic α-GalCer administration.

### FFD increases hepatic IL-12 production, a cytokine that reduces NKT cell viability

The differentiation and viability of NKT cells are regulated by cytokines. Previous studies have demonstrated that IL-12 significantly reduces NKT cell viability, while IL-15 increases NKT cell viability. Our subsequent efforts were directed to determine the mechanisms by which dietary factors might reduce hepatic NKT cells. The data showed that hepatic production of IL-12 was increased approximately 8-fold by FFD in WT mice than controls (Figure 4(E)) but unchanged by the MCD diet. Repeated injection of α-GalCer elevated hepatic levels of IL-12 by 4-fold in the MCD group but had no effect in the FFD group (Figure 4(E)). The hepatic level of IL-15 was unchanged by both the FFD and MCD diets (1.9 *vs*. 1.1, *p* = .100; 5.9 *vs*. 1.1, *p* = .051) (Figure 4(F)). The above results suggested that an increase in IL-12 may contribute to the significant depletion of NKT cells in mice.

### CD1d deficiency plays a role in the MCD-induced hepatic profibrogenic response but has little effect on FFD-induced hepatic fibrogenesis

**Figure 5.**
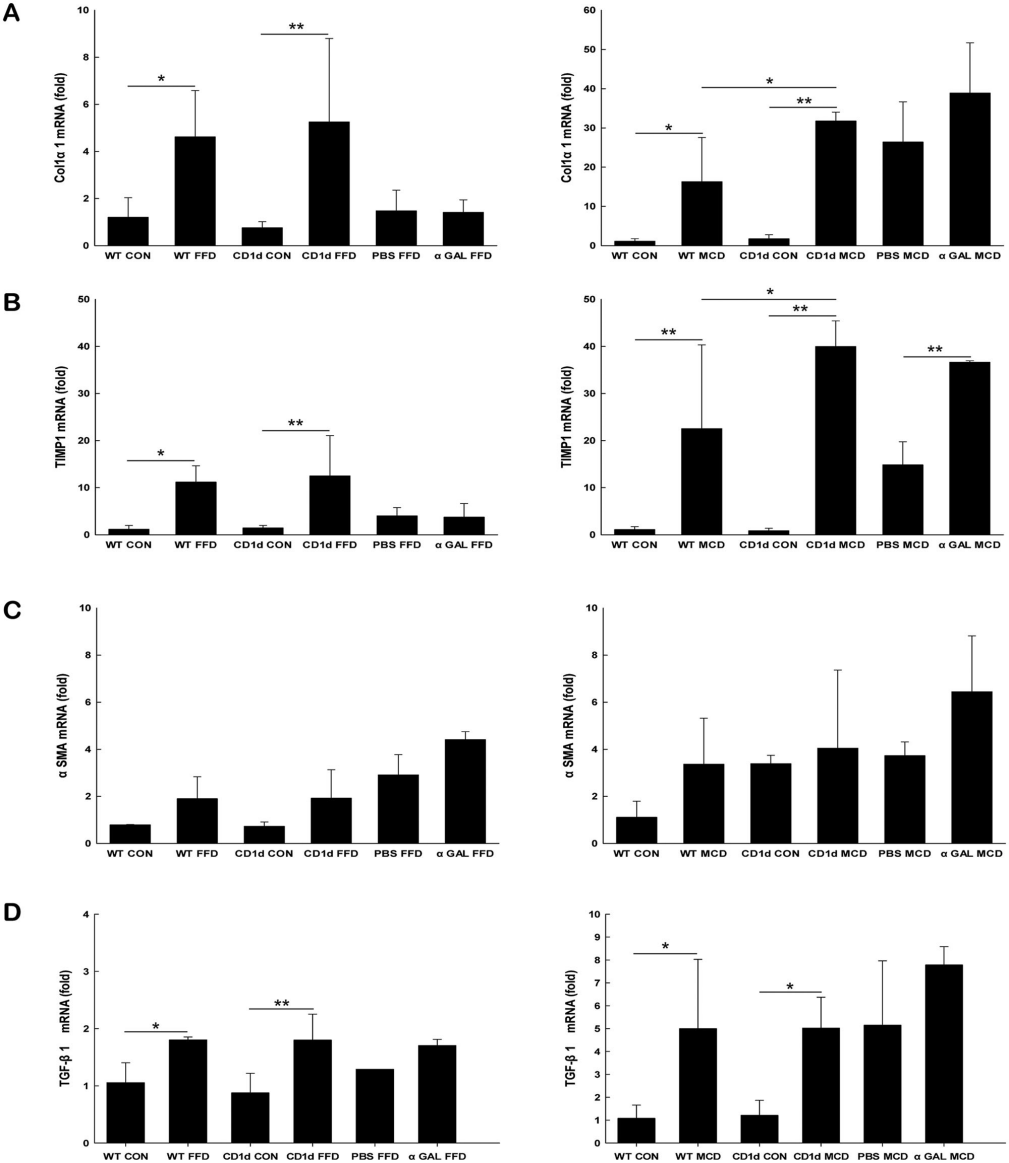
Either CD1d-deficiency or chronic α-GalCer treatment plays a role in MCD-induced hepatic profibrogenic responses The mRNA expression of hepatic Col1α1 (A), TIMP1 (B), α-SMA (C) and TGF-β1 (D) was measured by RT-qPCR. The data are presented as mean ± SD (*n* = 5 per group). *p < .05, **p < .01.

To extensively evaluate the effect of NKT cells on NASH-related fibrogenesis at the molecular level, either the FFD or MCD diet was administered to CD1d-/- mice and WT controls. Livers were then harvested for assessment of Col1α1, TIMP1, a-SMA and TGF-β1 gene expression. The hepatic mRNA expression of Col1α1 (Figure 5(A)) and TIMP1 (Figure 5(B)) was largely elevated in CD1d-/- mice than WT mice fed the MCD diet (31.8 *vs*. 16.3, *p* = .039; 40.0 *vs*. 22.6, *p* = .019), while the mRNA levels of a-SMA (Figure 5(C)), a marker of hepatic stellate cell activation, and TGF-β1 (Figure 5(D)) were not significantly changed in CD1d-/- mice compared to WT mice fed the MCD diet (4.1 *vs*. 3.4, *p* = .829; 5.0 *vs*. 5.0, *p* = .988). The hepatic mRNA expression of Col1α1 (Figure 5(A)), TIMP1 (Figure 5(B)), a-SMA (Figure 5(C)) and TGF-β1 (Figure 5(D)) was insignificantly changed in CD1d-/- mice compared to WT mice given FFD (5.3 *vs.* 4.6, *p* = .644; 12.5 *vs*. 11.2, *p* = .720; 1.9 *vs*. 1.9, *p* = .974; 1.8 *vs*. 1.8, *p* =.990). Consistent with the changes in ALT levels (Figure 1(E)) and hepatic relative fibrosis density (Figure 1(F)), these results indicated that CD1d deficiency had a role in the MCD-induced hepatic fibrogenic response at both the molecular and porphological level, while it had little effect on FFD-induced hepatic fibrogenesis.

### Chronic α-GalCer treatment plays a role in MCD-induced hepatic fibrogenesis at the molecular level

As mentioned earlier, the cytokine profile of NKT cells influences subsequent inflammatory responses of other leukocytes and hence a variety of pathological processes. To investigate the role of α-GalCer in NASH-related fibrosis, we examined the effects of repeated α-GalCer injection on fibrotic parameters in mice fed either an FFD or MCD diet. The results showed that α-GalCer-treated mice developed similar histological changes, such as steatosis, inflammation, and fibrosis, compared to their FFD or MCD control groups (Figure 1(C,D)). Morphometric analysis demonstrated that the relative fibrosis quantity in FFD- or MCD plus α-GalCer-treated mice was comparable to that in their control mice (Figure 1(F)). Only hepatic mRNA expression of TIMP1 was obviously increased in MCD plus α-GalCer-treated mice compared to control mice (36.7 *vs*. 14.9, *p* = .005) (Figure 5(B)), which suggests that NKT cell anergy or tolerance induced by chronic α-GalCer administration contributes to the profibrogenic molecular response in MCD-induced NASH.

## Discussion

It has been increasingly recognised that the immune response plays a key role in the pathogenesis of obesity-associated metabolic diseases, including NAFLD. The results focussing on either the pathogenic or protective role of NKT cells in the pathogenesis of NAFLD are controversial [21–24]. A number of studies have demonstrated that mice lacking NKT cells are susceptible to MS, steatosis, inflammation, and liver fibrosis during HFD or choline-deficient feeding [25–27]. However, other groups have indicated that NKT cells play a role in promoting liver fibrogenesis [13–15].

Among the various environmental factors that might contribute to obesity-related diseases, dietary habits merit particular consideration. It is therefore attractive to use dietary animal models to study disease pathogenesis. In our study, mice fed MCD diets for 8 weeks showed apparently severe paracinar steatosis, lobular and portal inflammation, and stage 2 or 3 fibrosis, which is consistent with previous studies [16]. After feeding FFD for 24 weeks, the mice showed microvesicular and macrovesicular steatosis, intraacinar inflammation and stage 1 fibrosis. In contrast to this study, Laura *et al.* reported that in another high fat and high fructose western diet (American Lifestyle-Induced Obesity Syndrome, ALIOS), only increased procollagen α1 mRNA abundance was observed, suggesting an early stage of fibrosis at the molecular level except for steatosis and inflammation [28]. Based on our study, FFD may induce early-stage fibrosis, while the MCD diet causes progressive liver fibrosis.

NKT cells represent an important link between innate and adaptive immunity through the production of Th1- and Th2-type cytokines. Previous studies reported reduced hepatic NKT cells in HFD-induced hepatosteatosis [8,10,11,27,29]. Hepatic NKT cell depletion is associated with reduced hepatic expression of antiinflammatory cytokines and increased proinflammatory cytokines, leading to the progression of NAFLD. Adoptive transfer of WT NKT cells led to improvement in glucose tolerance and steatosis [10,12]. In the current study, the proportion of hepatic NKT cells was significantly decreased in mice fed FFD but insignificantly changed in those fed MCD diets. Both the FFD and MCD diets increased hepatic proinflammatory cytokine production. These results suggested that NKT cells may have a protective role in the inflammatory responses of NAFLD. In contrast to the insignificant change in hepatic NKT cell abundance in MCD mice, Syn *et al.* demonstrated an obvious accumulation of NKT cells in the MCD model [13]. The discrepancy between the two studies is still uncertain, and we speculated that it may attributed to environments in animal facilities, mouse strains, different diet contents and time courses of treatment. Consistent with previous findings [30], we found that hepatic CD4^+^NKT cells were the predominant hepatic NKT cell fraction depleted, while double-negative (DN, CD4^-^/CD8^-^) NKT cells were relatively increased only in our results. The mechanism underlying the depletion of hepatic NKT cells in diet-induced NAFLD remains obscure. A previous study showed that high dietary fatty acids, such as saturated fatty acids and monounsaturated fatty acids, may impair the ability of CD1d to present endogenous antigens to hepatic NKT cells and contribute to NKT cell depletion, leading to liver steatosis and further activation of the inflammatory response [31]. Moreover, Tang *et al.* reported that a HFD increases KC numbers and their proinflammatory status, which subsequently causes hepatic NKT cell overactivation and cell death, leading to hepatic NKT cell deficiency in the development of NAFLD [32]. Findings from the current study indicated that hepatic IL-12, a cytokine that reduces NKT cell viability [33], was markedly increased in FFD mice, while it was insignificantly changed in MCD mice, which may partly account for the depletion of NKT cells in FFD WT mice and its maintenance in MCD WT mice.

While hepatic NKT cells have a protective role in hepatosteatosis, the role of these cells in advanced NASH-related fibrosis is still unclear. In the present study, FFD-induced hepatic fibrosis was comparable in WT mice and CD1d-deficient mice, while the MCD diet induced a higher degree of fibrotic response in CD1d-/- mice than WT mice, which suggested that NKT cells play a role in inhibiting liver fibrogenesis in the MCD model. The different effects of NKT cells on the fibrotic response in the two models may occur because hepatic NKT cells were significantly depleted in FFD mice but insignificantly changed in MCD mice. FFD induced remarkable depletion of hepatic NKT cells in WT mice. Thus, the fibrotic response in WT mice was very similar to that of CD1d-/- mice, as hepatic NKT cells were depleted in both groups. Our results and previous studies [31,32] indicate that NKT cells seem to play a protective role not only in hepatic steatosis but also in the progression of NAFLD.

α-GalCer, a glycolipid originally derived from a marine sponge, is an archetypical ligand for NKT cells. It is well established that α-GalCer causes a variety of responses in NKT cells, from the early activation phase with cytokine production to the subsequent tolerant state. Upon activation, NKT cells rapidly produce large quantities of Th1 and Th2 cytokines such as IFN-γ, TNF-α, IL-2, IL- 4, IL- 5, IL- 6, IL- 10, IL- 13, IL- 17, IL- 21, IL- 22, TGF-β and GM-CSF, which can influence differentiation, polarisation, and activation of a broad spectrum of immune cells, including dendritic cells (DC), macrophages, neutrophils, NK cells, and T and B cells [34,35]. Repeated a-GalCer injections biases DC maturation towards a tolerogenic phenotype in an IL-10 dependent manner [36]. Furthermore, NKT cells become unable to produce IFN-γ and IL-17 but persistent low IL-4 production [36]. On the one hand, NKT cell tolerance could serve as a model for the frequently observed NKT cell hyporesponsiveness in tumour and might help to develop strategies for their reactivation [37–39]. On the other hand, approaches to render NKT cells hyporesponsive may constitute new therapeutic strategies for diseases, where aberrant NKT cell activation is causally involved [40,41].

There is cumulating evidence that NKT cell activation have a pathogenic effect on a series of liver disorders, such as chronic hepatitis C [42], primary biliary cirrhosis [43]. Also, injection of α-GalCer induces a murine model of immune-mediated hepatitis which resembles human autoimmune-like liver disorders [44]. Findings from animal models demonstrated that a single α-GalCer injection may induce NKT cell activation, cytokine responses and liver injury [44–46]. However, repeated injection of α-GalCer may cause NKT cell anergy, tolerance, and imbalance of the Th1/Th2 cytokine profile [30,47]. Our data indicated that chronic administration of α-GalCer resulted in a significant decrease in hepatic NKT cells in both FFD and MCD mice. We demonstrated that chronic administration of α-GalCer enhanced hepatic Th2 cytokines in both FFD and MCD mice. Chronic administration of α-GalCer played a role in the MCD-induced hepatic profibrogenic response. In FFD WT mice, chronic administration of α-GalCer had minor effect on the liver fibrotic response, which may be attributed to the depletion of NKT cells in this model. Similar hyporesponsive effects of α-GalCer on liver fibrosis were also found in chronic CCL4-induced liver injury [48]. Thus, the hyporesponsiveness to α-GalCer treatment in FFD mice and its molecular responsiveness in MCD mice may be explained by the lack or relatively sufficient hepatic NKT cells during chronic liver inflammation in these models.

In summary, NKT cells may play a protective role in inhibiting diet-induced NAFLD. On the one hand, NKT cells are depleted in the FFD model, leading to a hepatic proinflammatory response and subsequent fibrosis. On the other hand, relatively sufficient NKT cells in the MCD model have a protective role in inhibiting the liver fibrotic response. We cannot elucidate at present the mechanism of differences in hepatic NKT cell abundance in early or advanced stages of diet-induced NAFLD in mouse models. We speculate that an increase in IL-12 and/or a decrease in IL-15 may contribute to this effect. NKT cells may be deficient during the early stage of fibrosis. After more extended period of disease, a shift in the hepatic microenvironment, such as the cytokine profile and chemokines, may support the recruitment or reconstitution of NKT cells. These issues warrant further investigation in order to better understand the underlying mechanisms.

## Data Availability

The data that support the findings of this study are available from the corresponding author, Dr. Shumei Zheng, upon reasonable request.
